# Fabrication and characterization of jujube extract‐loaded electrospun polyvinyl alcohol nanofiber for strawberry preservation

**DOI:** 10.1002/fsn3.2601

**Published:** 2021-09-23

**Authors:** Tayyebeh Zeinali, Esmat Alemzadeh, Asghar Zarban, Mohsen Khorashadizadeh, Elham Ansarifar

**Affiliations:** ^1^ Social Determinants of Health Research Center Department of Public Health School of Health Birjand University of Medical Sciences Birjand Iran; ^2^ Cellular and Molecular Research Center Department of Biotechnology School of Medicine Birjand University of Medical Sciences Birjand Iran; ^3^ Cardiovascular Diseases Research Center Department of Clinical Biochemistry School of Medicine Birjand University of Medical Sciences Birjand Iran

**Keywords:** electrospinning, fruit preservation, jujube extract, nanofiber, polyvinyl alcohol

## Abstract

Recently, using of natural ingredients gains much attention in the field of food science and active packaging. In this study, first, jujube extract was investigated for its antimicrobial and antioxidant properties, and then, the effect of electrospun PVA/JE (jujube extract loaded into Poly vinyl alcohol) nanofiber as active packaging was evaluated to increase the shelf‐life of strawberry. PVA/ZE nanofiber film was prepared using electrospinning method, and their morphology was confirmed by scanning electron microscopy (*SEM*). Fruit preservation abilities of the nanofiber film were tested on strawberries. The strawberries were then kept at 4℃ for 15 days and characterized in terms of their properties (weight loss, TSS, firmness, and sensory analysis). Results indicated that flavonoid content of jujube extract ranged from 4.80 ± 0.01 to 13.54 ± 0.08 mg CEQ/100 g, and the DPPH free radical‐scavenging activity was from 210 ± 2.66 to 1498 ± 2.65 (GAE/g DW). The jujube extract also presented potent antibacterial activity against the investigated bacteria and fungi. The scanning electron microscopy (*SEM*) images of nanofibers had a linear morphology and bead‐free structure; however, PVA/JE (jujube extract encapsulated into PVA nanofiber) had strip and flat organization. Strawberries in control group showed signs of decay and a decrease in visual appearance on the 6th. However, fruits in PVA/JE group had acceptable overall appearance for marketing, as no obvious sign of decay was observed on 12th day of storage. Active packaging containing herbal extracts and essential oils preserves the organoleptic and physicochemical properties of the fruits.

## Graphical abstract

In this study, jujube as an excellent source of antioxidant and antimicrobial component loaded into Poly vinyl alcohol (PVA) nanofiber by electrospinning for strawberry preservation.

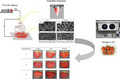

## 1 INTRODUCTION

Today, consumers prefer foods that use fewer treatments, including fewer preservatives with a more storage time (Ardekani et al., 2019). Active packaging as a response to this demand is a system to maintain and even improve the health properties, extending shelf‐life of food products, reduction of food waste, and wider distribution from the point of origin (Shahbazi, 2018). The mechanism of active packaging is that a small packet, envelope, or label is placed inside the packaging of the product (Li et al., 2020). These packets can contain antimicrobial, taste‐absorbent, and smell‐absorbent, enzymatic or antioxidant. This can release, such as, an antimicrobial substance to decline the process of deterioration, or can capture oxygen and moisture to ensure the food does not go off (Estevez‐Areco et al., 2018; Zanetti et al., 2018). Previous studies discovered that essential oil and herbal extract have significant antibacterial, antioxidant, anti‐parasitic, antifungal, and insecticidal activities (Shahbazi, 2018; Y. Zhang et al., 2019a). They are classified as Generally Recognized as Safe (GRAS) substances, and they are suitable alternatives to postharvest chemical preservatives (Dhital et al., 2018). However, due to high volatility and intense smell, use of them is limited in food packaging.

Encapsulation is a process to improve the physicochemical and microbiological stability of essential oils and herbal extract against environmental degradation. It controlled the rates of their release in the packaging (Zanetti et al., 2018). One of powerful methods for encapsulation, electrospinning, is mostly used for preparing polymer nanofibers and the encapsulation of bioactive component (Wen et al., 2017). The application of electrospun nanofibers in the food packaging has enormous attraction because of high surface area to volume, low cost, simple production, and large porosity (Lan et al., 2019). Poly vinyl alcohol (PVA) is one of polymers of food and drug administration (FDA) approved. It is utilized in the packaging industry due to its unique characteristics like film‐forming ability, biodegradability, low environmental impact, gas barrier performance, and low cost (Ardekani et al., 2019; Hadipour‐Goudarzi et al., 2014). The electrospun PVA nanofibers have many applications, including food packaging material, coating agent for food supplements, and pharmaceutical and cosmetics industrial use (Ardekani et al., 2019; Hadipour‐Goudarzi et al., 2014). However, being short of bactericidal properties of PVA limits the wider application in food packaging.

Jujube (*Ziziphus jujube* Mill.) belongs to the plant family *Rhamnaceae*. It is mainly distributed in the subtropical regions of Asia (south Khorasan province) (Afroz et al., 2014; Arab et al., 2017). It has been widely used due to its very beneficial effects on human health (Daneshmand et al., 2013). The highest components of jujube fruits are phenolic and flavonoids, which have antioxidant, antimicrobial, immune‐enhancing, anti‐inflammatory, gastrointestinal protective, and neuroprotective bioactivities (Afroz et al., 2014; Arab et al., 2017).

Until now, no study has reported the effects of incorporation of jujube extract in PVA electrospun nanofibers for quality maintenance of fruits and vegetables. So, first, the antimicrobial and antioxidant properties of jujube extract were investigated. Then, the electrospinning technique was used to fabricate nanofiber and encapsulation jujube extract into PVA nanofiber for food preservation. Application of PVA/JE on fruit shelf‐life was evaluated by using strawberry.

## 2 MATERIALS AND METHOD

### 2.1 Characterization of jujube extract

#### 2.1.1 Preparation of plant extracts

Fresh fruits of jujube were harvested from Herbarium of Birjand University, South Khorassan, Iran from September to October, 2019. These were dried and then ground with a grinder ( LGJM‐110, china). The aqueous extract of jujube was prepared as follows: add 10 g of powder in 100 ml of water and stir for 24 h, and then, the mixtures were filtered through a No. 1 Whatman filter paper. Eventually, the samples lyophilized by freeze dryer (Dena, model: FD‐50F series, Iran) at −80℃ for 24 h (Safizadeh et al., 2017). For determination of antioxidant activity and total phenol and flavonoid contents, 5, 10, 15, and 20 g/ml concentrations were used.

#### 2.1.2 Measurement of total flavonoids content

The flavonoid content in the jujube extract was analyzed according to the aluminum chloride colorimetric method. The concentration of the total flavonoid was determined as catechin equivalents, and the results were expressed as mg of CEQs per 100 g (Afroz et al., 2014).

#### 2.1.3 DPPH assay

Scavenging activity of 2,2‐diphenyl‐1‐picrylhydrazyl (DPPH) radicals was measured according to the method described by (Arab et al., 2017; Hemmati et al., 2015).

#### 2.1.4 Total phenol evaluation by Folin–Ciocalteu assay

A Folin–Ciocalteu procedure was used to measure the total phenolic content of JE. Phenolic and polyphenolic contents were determined as a Gallic acid equivalents per gram of dry material (Olajuyigbe & Afolayan, 2011).

#### 2.1.5 Antimicrobial activity of jujube extract

The antimicrobial activity of aqueous extract of jujube was investigated using the following bacteria: *Staphylococcus aureus* (ATCC 29,213) and *Pseudomonas aeruginosa* ATCC 13,525. Antifungal activity of the extract of jujube was tested on *Candida albicans*. In order to determine the minimum inhibitory concentration (MIC) as the lowest concentration in which the visible growth inhibited, a microbroth dilution assay was applied (Afroz et al., 2014). After the MIC determination, to determine Minimum bactericidal concentration (MBC), aliquots of 50 μl from all the tubes which showed no visible bacterial growth were seeded on BHI agar plates and incubated for 24 h at 37℃.

### 2.2 Characterization of nanofibers

#### 2.2.1 Solution preparation

The 10% (w/v) polyvinyl alcohol (PVA, Mw = 130,000 Da, Aldrich Chemical) solution was made at 80℃ and then stirred for 2 h until production of homogeneous solution. Jujube extract (5% w/v) was added to PVA solution (when it has reached ambient temperature) and mixed for 24 h.

#### 2.2.2 Electrospinning

Nanofibers were produced using Electrospinning with a high‐voltage instrument (ES‐1000, Nanoscale technologists CO., Iran) that positive and negative electrodes of high voltage were connected to the capillary needle, and rotating collector was wrapped with aluminum foil. The prepared solutions were loaded into a 10 ml syringe equipped with a stainless steel needle. Rate injection, voltage, and distance between the needle tip to collector were set at 0.5 ml/h, 15 kV, and 15 cm, respectively (Ardekani et al., 2019).

#### 2.2.3 Scanning electron microscopy (SEM)

Scanning electron microscope (Leo 1450VPSEM) with an acceleration voltage of 20 kV was used to observe the morphology of the nanofibers (Lan et al., 2019).

#### 2.2.4 Antibacterial activity of nanofiber

The antimicrobial activity of nanofiber against gram‐positive bacteria and gram‐negative bacteria was determined using disk diffusion method after production fibers. Film samples were cutted into disk shaped with diameter of 10 mm and then placed on Mullere Hinton Agar Plate (MHA) plates which had been already smeared with 1.5 × 10^8^ CFU/ml colonies of *Staphylococcus aureus* (ATCC 29,213) and *Pseudomonas aeruginosa* ATCC 13,525, and then incubated at 37℃ for 24 h (Dashipour et al., 2015).

### 2.3 Application nanofibers for active packaging

#### 2.3.1 Fruit preparation and active packaging

Strawberries were selected according to similarity of color, size, appearance, and the absence of microbial spoilage and surface defects. Fruits were washed and immersed in a solution of 0.1% sodium hypochlorite for 1 min and then dripped off for 5min. The fruits were divided into three groups and placed in polyethylene containers with dimension of (11.5 × 9.5 × 6.2 cm) and packed with its lid. Group 1(control): The fruit packed in PET container without film on its lid, group 2 (PVA): The fruit packed in PET container with PVA nanofiber film on its lid, and group 3 (PVA/JE): The fruit packed in PET container with jujube extract loaded into PVA nanofiber film on its lid. Packages of fruit were kept in a cold room at 4 ± 0.5℃ and 85% RH for 12 days. Physicochemical properties of strawberries were evaluated at different time intervals at day 0, 3, 6, 9, and 12 of storage.

### 2.4 Assessment of strawberry quality

#### 2.4.1 Weight loss

Fruits were weighed by a digital balance (UWA‐K‐015, China). The result was calculated as the percentage of the total weight loss between the initial and final weight (Noshad et al., 2015).

#### 2.4.2 Total soluble solids (TSS)

Fruit juice was collected by juicer (Pars khazar, model: JBG‐610SP, Iran). The TSS of juice was evaluated by a hand‐held refractometer (Atago, Tokyo, Japan) at 20℃ (Shahbazi, 2018).

#### 2.4.3 Firmness measurements

The firmness of the strawberries was determined using a digital penetrometer (FHT200, Extech Co., USA), fitted with a 2 mm diameter cylinder probe.

#### 2.4.4 Sensory analysis

Sensory analysis of samples was done by a panel of eight trained members (four men and four women), based on a 9‐point hedonic scale on the 0, 6, and12th day of storage. Panelists rated the color, texture, taste, visual appearance, and general acceptance of strawberries on a five‐point scale, 1 = extremely bad and 9 = extremely good (4.5 and higher =acceptable) (Wen et al., 2017).

### 2.5 Statistical analysis

The difference between samples was evaluated with analysis of variance (one‐way ANOVA with Duncan test) by p ˂ .05 as significance level and post hoc test for independent samples by using the statistical software SPSS (IBM SPSS Statistics, Version 22, New York, USA). Notably, the experiments were conducted in triplicate.

## 3 RESULT AND DISCUSSION

### 3.1 Characterization of jujube extract

#### 3.1.1 Antioxidant activity and total flavonoids and phenolic content

Flavonoids are natural phenolic compounds which have a wide range of pharmacological and biochemical properties (Topuz & Uyar, 2019). As is seen in Table 1, flavonoid content of jujube extract in different concentration ranged from 4.80 ± 0.01 to 13.54 ± 0.08 mg CEQ/100 g and also, and total phenolic content (TPC) was from 184 ± 23.12 to 990 ± 54.11 GAE/100 g dry weight of plant material. (Table 1). Afroz et al. (2014) and Koley et al. (2016) reported the estimated flavonoid content of Apple kul (Zizyphus Mauritiana) and Indian Zizyphus as (13.19 ± 1.31 mg CEQ/100 g) and (13.09 ± 3.93 mg CEQ/100 g), respectively, which was similar to our study values (Afroz et al., 2014; Koley et al., 2016). It was reported that total phenolic content of jujube (2.5 g/L) was 220 ± 2.76 GAE/g DW (Safizadeh et al., 2017). Hudina et al (2014) found that extract of Chinese jujube contained two phenolic acids (chlorogenic acid and caffeic) and three flavonoids (catechin, epicatechin, and rutin) (Hudina et al., 2014). DPPH is a stable free radical that is used to determine radical‐scavenging activity of natural compounds (H. Zhang et al., 2010). Thus, the radical‐scavenging activity in the presence of a hydrogen‐donating antioxidant can be monitored as a decrease in absorbance of DPPH solution (Afroz et al., 2014). Table 1 showed free radical‐scavenging activity of the jujube extract at different concentration. The DPPH free radical‐scavenging activity was from 210 ± 2.66 to1498 ± 2.65 (GAE/g DW).

**TABLE 1:** The flavonoid and phenolic content and DPPH scavenging activity of different concentration of jujube extract

Content	Different concentration of jujube extract				
	2.5	5	10	15	20
Flavonoid content (mg CEQ/100 g DW)	4.80 ± 0.34^d^	7.45 ± 0.12^c^	9.02 ± 1.02^bc^	10.42 ± 2.06^b^	13.54 ± 2.08^a^
DPPH scavenging activity (GAE/g DW)	210 ± 52.66^d^	563 ± 63.21^bc^	850.3 ± 57.31^b^	1,317 ± 72.32^ab^	1,498 ± 76.65^a^
Total phenolic content (GAE/g DW)	184 ± 23.12^e^	345 ± 89.21^d^	503 ± 81.65^c^	743 ± 98.12^b^	990 ± 103.11^a^

Note

Data shown are the mean ±standard error of three replicates. ^a‐e^ Different letters indicate significant difference for the concentration of jujube extract.

As the results showed, there was a correlation between phenolic and flavonoid content with antioxidant capacity. Gao et al (2013) and Zhang et al. (2010) reported similar results about relationship between total phenolic content and high antioxidant activity.

The scavenging activity of jujube extract on inhibition of the DPPH radical was related to the concentration of extract added. This result agreed with Arab et al. (2017) that reported the scavenging effects on the DPPH radical increase sharply with the growing concentration of the samples to a certain extent (Arab et al., 2017).

### 3.2 Antimicrobial activity

Plant extracts show significant antimicrobial activities due to the presence of different phenolic compounds and the flavonoid contents (Afroz et al., 2014). The jujube extract presented potent antibacterial activity against the investigated bacteria and fungi (Table 2). *P. aeruginosa* is a gram‐negative bacterium that is a major causative agent for lung infections or pneumonia (Daneshmand et al., 2013). Jujube extract had shown activity against *P. aeruginosa* (MIC: 2.76 ± 0.32 mg/ml). *S. aureus* is a gram‐positive bacteria generally found on the skin and mucus membranes (Daneshmand et al., 2013). The MIC of jujube extract against *S. aureus* was 4.26 ± 0.68 mg/ml. MIC value of jujube extract against *C*. *albicans* was 3.35 ± 0.38 mg/ml (Table 2). These MIC values were similar with many other medicinal plants; for example, Santos et al. (2008) reported MIC value of essential oil extracted from leaves of *Hyptis pectinata* against *P. aeruginosa* as 200 mg/ml (Santos et al., 2008). Afroz et al. (2014) stated MIC value of the methanolic extract of garlic root against *S. aureus* as 100 mg/ml (Afroz et al., 2014). Overall, the results showed that jujube extract had inhibitory effect on studied bacteria and fungi and it has the potential to be used against *P. aeruginosa* and *S. aureus* infections. This is due to the presence of phenolic compounds and the flavonoid contents in jujube extract which was confirmed by antioxidant activity and total flavonoids content (Table 1).

**TABLE 2:** Antimicrobial activity (MIC/MBC) of the aqueous extract of jujube and inhibition zone of nanofiber (PVA and PVA/JE)

Microbe	Antimicrobial activity of JE		Inhibition zone of nanofiber (mm)	
	MIC (mg/ml)	MBC (mg/ml)	PVA	PVA/JE
*Staphylococcus aureus*	4.26 ± 0.68	5.1 ± 0.31	0	11.93 ± 0.32
*Pseudomonas aeruginosa*	2.76 ± 0.32	2.86 ± 0.63	0	7.43 ± 0.71
*Candida albicans*	3.35 ± 0.38	4.54 ± 0.21	0	‐

Note

Data shown are the mean ±standard error of three replicates.

### 3.3 Characterization of nanofibers

According to the results of antimicrobial and antioxidant activity of jujube extract, concentration of 5% was selected for loading in PVA nanofiber The encapsulation efficiency was about 88.34%. The release rate of JE encapsulated in PVA nanofiber film was much slower than free JE. 53% of encapsulated JE was released after 72h.

### 3.4 SEM

**FIGURE 1:**
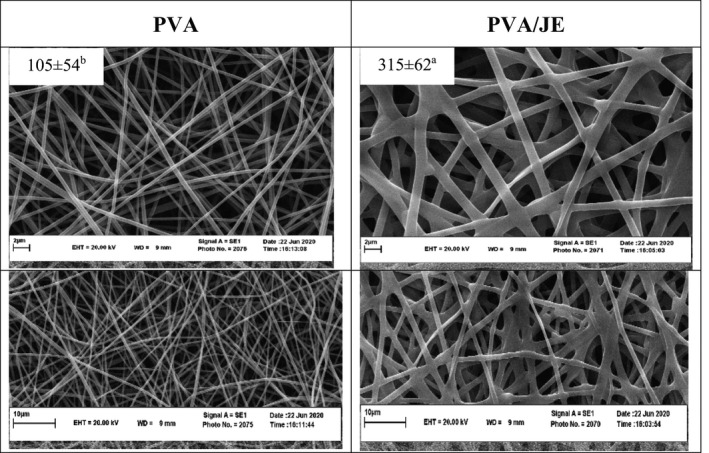
*SEM* image of PVA and PVA/JE nanofibers, (mean±*SD*; mean with different letters are statistically significant; p ˂ .05)

The *SEM* images and diameter distribution of PVA and PVA/JE nanofiber are presented in Figure 1. The fiber average diameter of PVA and PVA/JE films (*n* = 30) was 105 ± 54 and 315 ± 62 nm, respectively. Wen et al. (2016) reported average diameter of electrospun PVA/cinnamon essential oil/β‐cyclodextrin (PVA.CEO/β‐CD) antimicrobial nanofibrous as 240 ± 40 nm (Wen et al., 2016). As seen in Figure 1, all nanofibers had a linear morphology, smooth surface, and bead‐free structure; however, PVA/JE nanofibers were strip and flat. Average diameter of PVA/JE nanofiber film was significantly (p˂0.05) higher than PVA nanofiber film. This is probably due to herbal extract encapsulation into matrix of film, reducing electrical conductivity of solution and then decreasing in the elongation of polymer jet by the applied voltage, so, diameter of nanofiber increases. This result was confirmed by Li et al. (2020) and Wen et al. (2016) that encapsulated eugenol into PVA/shellac fibrous films and cinnamon essential oil into PVA/β‐cyclodextrin, respectively (Li et al., 2020; Wen et al., 2016).

### 3.5 Antibacterial activity of nanofiber

Inhibition effect of antimicrobial PVA/JE nanofiber on foodborne pathogen was investigated in vitro. Inhibition zone of PVA and PVA/JE nanofiber against *Staphylococcus aureus and Pseudomonas aeruginosa* showed in Table 2. As is known, the PVA nanofiber film as control group does not exhibit any antimicrobial properties without aseptic region, while apparent inhibition rings are detected around PVA/JE. Inhibition zone of PVA/JE nanofiber film against Staphylococcus *aureus and Pseudomonas aeruginosa* was 11.93 ± 0.32 and 7.43 ± 0.71, respectively. Gram‐negative bacteria were more resistant than Gram‐positive bacteria. Main mechanism for the antibacterial effect of essential oils and herbal extracts is their attacking the phospholipid bilayer of bacterial cell membrane which causes morphological damages and disrupts membrane integrity of bacteria also disrupting their enzyme systems. These results are in agreement with the reports of some studies that stated essential oil and herbal extracts significantly interrupted the cell membranes of some bacteria such as Escherichia coli O157 H7, S. aureus, and S. Typhimurium (Dashipour et al., 2015; Nazari et al., 2019; Zhang et al., 2019).

### 3.6 Fruit physicochemical measurements

#### 3.6.1 Weight loss

Water loss of fruits and vegetables is due to respiration and transpiration, which leads to reduce their freshness and commercial value (Shahbazi, 2018). As seen in Table 3, the fruits’ weight loss gradually increased during storage due to its respiratory process. Weight loss of all fruit treatments was particularly pronounced from day 6 onwards. It can be due to some strawberries started to decay on day 6, mostly the control group. The lowest weight loss (11.21 ± 0.13) was obtained in strawberries packed with PVA/JE nanofiber at day 3 of storage (Table 3), while the highest weight loss (22.67 ± 0.32) was observed in control samples at day 12 of storage. This was because of the eventual accumulation of moisture produced by the respiration of strawberries inside the control package. This trapped moisture created an environment that was conducive for fungal growth. In the event that, in the PVA and PVA/JE packages, moisture from the respiration of fruit was absorbed by the nanofiber film. Also, compounds of jujube extract released from the nanofiber in PVA/JE treatment slow down the ripening process of treated fruit and thus significantly gave lower weight loss from day 6 to the end of storage period. Amal et al. (2010) also reported that treated strawberry fruits with thyme essential oil significantly reduced fruit weight loss compared with the control (Amal et al., 2010).

**TABLE 3:** Physicochemical properties (weight loss, TSS, firmness, and antioxidant capacity) of strawberries packed in packaging contain PVA nanofiber (PVA) and PVA nanofiber loaded with jujube extract (PVA/JE) during 12 days of storage at 4ºC

Parameter	Active packaging Treatment	Storage time (Day)				
		0	3	6	9	12
Weight loss (g)	Control	‐	1.91 ± 0.12^abC^	9.63 ± 0.31^aB^	20.15 ± 0.09^aA^	22.67 ± 0.32^aA^
	PVA	‐	2.20 ± 0.25^aC^	8.23 ± 0.25^bB^	14.84 ± 0.21^bA^	15.63 ± 0.42^bA^
	PVA/JE	‐	1.59 ± 0.04^bC^	5.45 ± 0.43^abB^	8.02 ± 0.23^cA^	11.21 ± 0.13^cA^
TSS	Control	3.14 ± 0.12^aBC^	5 ± 0.34a^A^	3.71 ± 0.23^bB^	2.85 ± 0.15^bC^	2.65 ± 0.11^cCD^
	PVA	3.21 ± 0.10^aC^	4.22 ± 0.12^bA^	3.92 ± 0.14^aB^	2.95 ± 0.08^bD^	2.97 ± 0.21^bD^
	PVA/JE	3.13 ± 0.09^aC^	4.47 ± 0.11^abA^	3.97 ± 0.23^aB^	3.12 ± 0.13^aC^	3.15 ± 0.14^aC^
Firmness (*N*)	Control	1.25 ± 0.07^aA^	0.76 ± 0.02^bB^	0.68 ± 0.03^cC^	0.63 ± 0.01^cC^	0.53 ± 0.01^cD^
	PVA	1.29 ± 0.01^aA^	0.96 ± 0.01^abB^	0.87 ± 0.02^bC^	0.72 ± 0.01^bD^	0.68 ± 0.02^bE^
	PVA/JE	1.23 ± 0.01^aA^	1.18 ± 0.04^aB^	1.08 ± 0.02^aC^	0.98 ± 0.03^aD^	0.84 ± 0.01^aE^
Antioxidant capacity	Control	28.02 ± 1.32^aD^	29 ± 2.45^aD^	40 ± 1.76^cC^	66.54 ± 2.54^cA^	62.5 ± 3.21^bcAB^
	PVA	26.02 ± 3.03^aC^	28.65 ± 1.87^aC^	53.5 ± 1.54^bB^	74.51 ± 2.43^bA^	68.5 ± 3.24^bAB^
	PVA/JE	25.02 ± 2.41^aC^	29.12 ± 2.65^aC^	59.12 ± 3.61^aB^	83.43 ± 3.44^aA^	80.5 ± 1.76^aA^

Note

Data shown are the mean ±standard error of three replicates. ^a‐e^ Different letters indicate significant difference between packaging treatments within each storage time. ^A‐E^ Different letters indicate significant difference in the storage time for each packaging treatment.

#### 3.6.2 Total soluble solids

The TSS of all treatments increased until the third day of storage. It could be because of hydrolysis of the cell wall and breakdown of starch into soluble sugars (Noshad et al., 2015). From the third day, the TSS of treatment started to decrease. Rate of decrease was faster in the control samples than in other samples, because the control group showed the highest respiration. After 12 days of storage, strawberries that were packed in the container with nanofiber PVA or PVA/JE had higher TSS compared with control samples (Table 3). It is likely due to the slowing down of respiration and reduction of sugars in the strawberries (Dong & Wang, 2017). Liu et al. (2019) reported the TSS of fruits that wrapped with PLA/CNTs/CS nanomaterials were lower than the unwrapped fruits. They suggested that the PLA/CNTs/CS nanomaterials slowed down the reduction of metabolic and hydrolyze sugars (Liu et al., 2019).

### 3.7 Firmness

Firmness is one of the important quality changes that occur during fruit storage. As seen in Table 3, firmness of all treatments gradually decreased during storage time. This can be due to the degradation of the cell wall and loss of water by the cell breakdown (Arabpoor et al., 2021). Fruits that are stored in nanofiber‐containing packages exhibited higher firmness compared with the control during 12 days of cold storage. The results suggest that jujube extract released from the PVA nanofiber did preserve better the cell wall integrity of fruit in PVA/JE treatment and reduced moisture loss. This result is similar to findings of (Hadipour‐Goudarzi et al., 2014) that observed strawberries wrapped in nanofiber had the best firmness during the 8‐day experiment which was related to the moisture loss in the packaging and avoiding carbon dioxide emission. Also, Wen et al. (2016) reported the samples packed with PVA/CEO/b‐CD nanofilm had higher firmness values than the others. The firmness of the control and the strawberries packed with fresh‐keeping film and PVA/CEO/b‐CD nanofilm was decreased to 53.3%, 34.8%, and 14.0%, respectively, at day 18 (Wen et al., 2016). In this study, being packaged with nanofibers preserved the firmness of strawberries, suggesting it could be used as a hopeful method to inhibit fruit softening.

### 3.8 Antioxidant capacity

Antioxidant capacity is an important parameter in evaluating fruits. The DPPH method was used to measure the antioxidant capacity of fruits, due to their sensitivity, validity, speed, and easiness (Topuz & Uyar, 2019). Total antioxidant capacity of strawberries at the three groups (control, packaging contain PVA, and PVA/JE nanofiber film) during 12 days of storage at 4℃ was shown in Table 3. Trend of antioxidant content of all treatments of strawberry was increased until 6 days of storage. After that, the antioxidant content of samples was decreased. The reduction of antioxidant capacity in fruits at the end of storage time might be due to senescence (Arabpoor et al., 2021). These findings were in agreement with the results of Dong and Wang. (2017) and Kamkar et al. (2021) that reported the edible coating incorporated with essential oil significantly improved the total antioxidant capacity of coated strawberries over uncoated fruits (Dong & Wang, 2017; Kamkar et al., 2021). However, the reduction in antioxidant content in strawberries packed in PVA/JE (from 25.02 ± 2.41 to 80.5 ± 1.76) and PVA (from 26.02 to 68.5 ± 3.24) nanofiber film was lower than the control (from 28.02 ± 1.32 to 62.5 ± 3.21) fruits at the end of storage time. The highest content of antioxidant was in fruits stored in packaging contain PVA/JE nanofiber. It result might be due to antioxidant compounds especially polyphenol of jujube extract added to strawberries after releasing of nanofiber film. Similarly, Arabpoor et al. (2021) stated that total antioxidant content of sweet cherries coated with chitosan nanoparticles containing essential oil was higher than the control during 21 days of storage (Arabpoor et al., 2021).

### 3.9 Sensory evaluation

**FIGURE 2:**
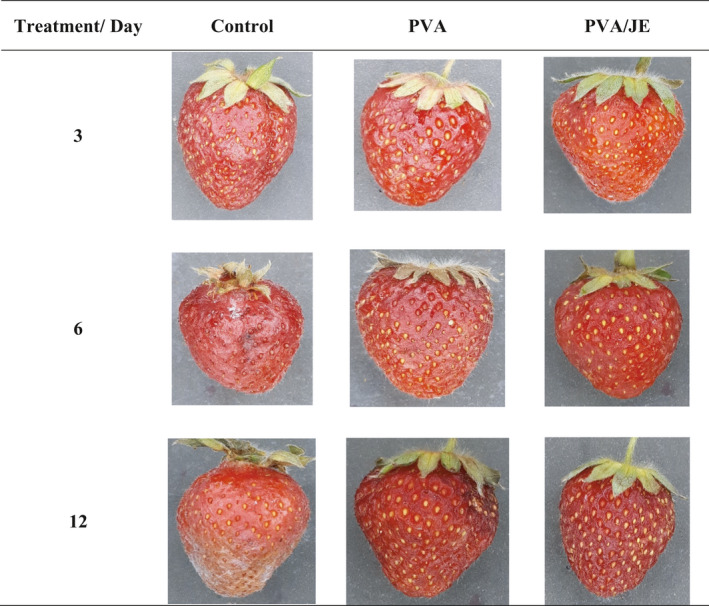
Overall appearance of strawberries packed in packaging contains PVA nanofiber (PVA) and PVA nanofiber loaded with jujube extract (PVA/JE) during 12 days of storage at 4℃

The sensory evaluation is necessary because the acceptability of treated fruits is determined by final consumer (Wen et al., 2017). Results of color, texture, taste, appearance, and general acceptance parameters of all samples at days 6th and 12th of storage are presented in Table 4 and Figure 2. As seen in Table 4, Sensory attributes were significantly affected by storage time in all treatments. Strawberries in the control group showed signs of decay and also a decrease in visual appearance on the 6th day of storage, resulting in this group had not general acceptance for consumption. However, fruits that were packed in PVA/JE nanofiber packaging had acceptable overall appearance for marketing, as no obvious signs of decay were observed at 12th day of cold storage (Figure 2). The taste of fruits was affected by treatment (control, PVA, and PVA/JE) and storage time. The panelist marked that treated strawberries had a slightly sour taste compared with control strawberries. This sensory result was in agreement with TS result (Table 4). At the end of storage, the scores of color and texture were higher in fruits treated with PVA/JE nanofiber film than control fruits. These results also confirmed results of pentameter analyzer and color processing (Table 3). This behavior shows that active packaging with herbal extracts and essential oils improves the organoleptic and physicochemical properties of the strawberries (Wen et al., 2017).

**TABLE 4:** Sensory attributes of strawberries packed in packaging contain PVA nanofiber (PVA) and PVA nanofiber loaded with jujube extract (PVA/JE) during 12 days of storage at 4ºC

Day/treatment		0	6	12
Color	Control	8.12 ± 0.15 ^aA^	5.34 ± 0.13 ^bB^	3.92 ± 0.13 ^bcC^
	PVA	8.18 ± 0.10 ^aA^	5.80 ± 0.12 ^bB^	4.62 ± 0.12 ^bC^
	PVA/JE	8.15 ± 0.07 ^aA^	7.35 ± 0.08 ^aB^	6.10 ± 0.11 ^aC^
Texture	Control	8.43 ± 0.09 ^aA^	5.21 ± 0.08 ^bB^	4.26 ± 0.04 ^cC^
	PVA	8.38 ± 0.12 ^aA^	7.10 ± 0.11^aB^	5.23 ± 0.07 ^bC^
	PVA/JE	8.42 ± 0.10 ^aA^	7.30 ± 0.21 ^aB^	6.27 ± 0.14 ^aBC^
Taste	Control	8.20 ± 0.13 ^aA^	‐	‐
	PVA	8.22 ± 0.08 ^aA^	6.32 ± 0.12 ^aB^	4.80 ± 0.15 ^bC^
	PVA/JE	8.18 ± 0.10 ^aA^	6.10 ± 0.07 ^aB^	5.10 ± 0.15 ^aBC^
Appearance	Control	8.42 ± 0.13 ^aA^	4.34 ± 0.12 ^bB^	3.56 ± 0.12 ^cBC^
	PVA	8.32 ± 0.12 ^aA^	6.10 ± 0.06 ^aB^	4.95 ± 0.11 ^bC^
	PVA/JE	8.45 ± 0.08 ^aA^	6.97 ± 0.07 ^aB^	6.30 ± 0.04 ^aBC^
General acceptance	Control	8.05 ± 0.08 ^aA^	4.87 ± 0.10 ^cB^	3.43 ± 0.08 ^cC^
	PVA	8.10 ± 0.13 ^aA^	6.43 ± 0.07 ^bB^	5.10 ± 0.11 ^bC^
	PVA/JE	8.13 ± 0.14 ^aA^	7.20 ± 0.02 ^aAB^	6.44 ± 0.06 ^aBC^

Note

Data shown are the mean ±standard error of three replicates. ^a‐e^ Different letters indicate significant difference between packaging treatments within each storage time. ^A‐E^ Different letters indicate significant difference for the storage time for each packaging treatment.

## 4 CONCLUSION

In this study, it was confirmed that the jujube extract had potent antibacterial and antioxidant activity. Also, it has shown the possibility of fabrication and application of PVA nanofiber‐containing JE. PVA/JE nanofiber displayed a significant effect on extending the shelf‐life and preserving the quality of strawberries during storage time. These results confirmed electrospinning method is an efficient technique to encapsulate bioactive compounds. In conclusion, this study demonstrated the benefits of loading JE into PAV nanofiber film, which might have a high potential performance in active packaging.

## AUTHOR CONTRIBUTIONS

**Tayebeh Zeinali:** Conceptualization (equal); Methodology (equal); Project administration (equal); Validation (equal); Writing‐review & editing (equal). **Esmat Alemzadeh:** Data curation (equal); Formal analysis (equal). **Asghar Zarban:** Formal analysis (equal); Investigation (equal); Methodology (equal). **Mohsen Khorashadizadeh:** Methodology (equal); Project administration (equal). **Elham Ansarifar:** Conceptualization (equal); Data curation (equal); Formal analysis (equal); Funding acquisition (equal); Investigation (equal); Methodology (equal); Project administration (equal); Software (equal); Writing‐original draft (equal); Writing‐review & editing (equal).
