# Correction to “Fabrication and Characterization of Jujube Extract‐Loaded Electrospun Polyvinyl Alcohol Nanofiber for Strawberry Preservation”

**DOI:** 10.1002/fsn3.71325

**Published:** 2025-12-06

Zeinali, T., Alemzadeh, E., Zarban, A., Khorashadizadeh, M., and Ansarifar, E. 2021. “Fabrication and Characterization of Jujube Extract‐Loaded Electrospun Polyvinyl Alcohol Nanofiber for Strawberry Preservation.” *Food Science & Nutrition*, 9: 6353–6361. https://doi.org/10.1002/fsn3.2601.

The fiber morphology data presented in the Results section and Figure 1 require the following corrections:

- In Section 3.4, “The fiber average diameter of PVA and PVA/JE films (*n* = 30) was 105 ± 54 and 315 ± 62 nm, respectively” should be corrected to: “The SEM images and diameter distribution of PVA and PVA/JE nanofibers are presented in Figure 1. The fiber average diameter of PVA and PVA/JE films (*n* = 30) was 165 ± 54 and 845 ± 62 nm, respectively.”
- In Figure 1, the numerical values indicating the average fiber diameter should be updated to 165 ± 54 nm and 845 ± 62 nm. The corrected figure appears below.

**FIGURE 1:**
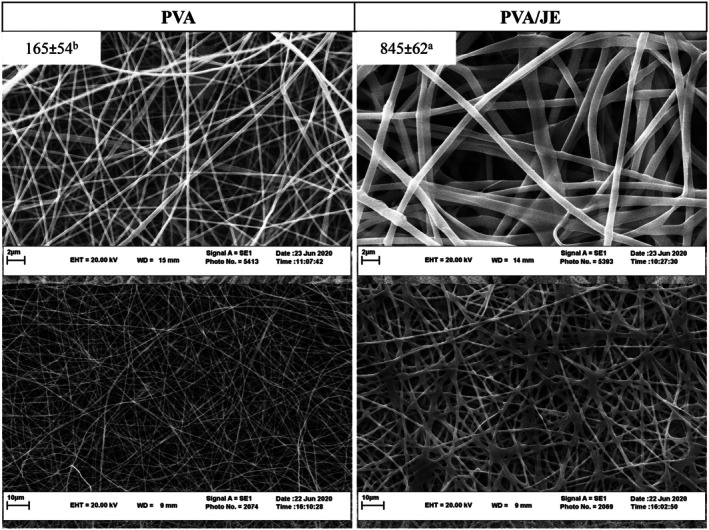
SEM image of PVA and PVA/JE nanofibers, (mean ± SD; mean with different letters are statistically significant; *p* < 0.05).

The originally published Figure 2 was a schematic representation of the visual aging trend and did not provide quantitative or comparative data that effectively support our experimental results. The corrected image offers higher visual quality and is more relevant to the data and discussion. The corrected figure appears below.

**FIGURE 2:**
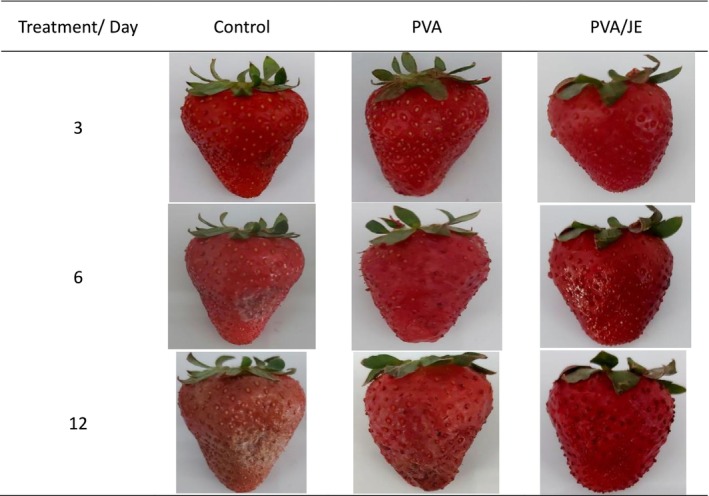
Overall appearance of strawberries packed in packaging contain PVA nanofiber (PVA) and PVA nanofiber loaded with jujube extract (PVA/JE) during 12 days of storage at 4°C.

We apologize for these errors.

